# Epidemiology of chlamydial infection and disease in a free-ranging koala (*Phascolarctos cinereus*) population

**DOI:** 10.1371/journal.pone.0190114

**Published:** 2017-12-27

**Authors:** Sharon Nyari, Courtney A. Waugh, Jianbao Dong, Bonnie L. Quigley, Jonathan Hanger, Joanne Loader, Adam Polkinghorne, Peter Timms

**Affiliations:** 1 Centre for Animal Health Innovation, Faculty of Science, Health, Education and Engineering, University of the Sunshine Coast, Sippy Downs, Queensland, Australia; 2 Endeavour Veterinary Ecology, Toorbul, Queensland, Australia; University of California, San Francisco, Universit of California, Berkeley and the Childrens Hospital Oakland Research Institute, UNITED STATES

## Abstract

Chlamydial disease continues to be one of the main factors threatening the long-term survival of the koala (*Phascolarctos cinereus*). Despite this, large epidemiological studies of chlamydial infection and disease in wild koala populations are lacking. A better understanding of the prevalence, transmission and pathogenesis is needed to improve control measures, such as the development of vaccines. We investigated the prevalence of *Chlamydia pecorum* infection and disease in 160 koalas in a peri-urban wild population in Queensland, Australia and found that 31% of koalas were *Chlamydia* PCR positive and 28% had clinically detectable chlamydial disease. Most infections were at the urogenital site (27%; both males and females) with only 14% at the ocular site. Interestingly, we found that 27% (4/15) of koalas considered to be sexually immature (9–13 months) were already infected with *C*. *pecorum*, suggesting that a significant percentage of animals are infected directly from their mother. Ocular infection levels were less prevalent with increasing age (8% in koalas older than 4 years), whereas the prevalence of urogenital tract infections remained high into older age (26% in koalas older than 4 years), suggesting that, after mother-to-young transmission, *C*. *pecorum* is predominantly a sexually transmitted infection. While 28% of koalas in this population had clinically detectable chlamydial disease (primarily urogenital tract disease), many PCR positive koalas had no detectable disease and importantly, not all diseased animals were PCR positive. We also observed higher chlamydial loads in koalas who were *C*. *pecorum* infected without clinical disease than in koalas who were *C*. *pecorum* infected with clinical disease. These results shed light on the potential mechanisms of transmission of *C*. *pecorum* in koalas and also guide future control measures, such as vaccination.

## Introduction

Chlamydial infection causes debilitating disease in the koala (*Phascolarctos cinereus*), threatening the long-term survival of this iconic Australian marsupial. It most commonly manifests as ocular and urogenital tract disease, however, disease may be subclinical and go undetected for long periods. Ocular infections can cause kerato-conjunctivitis which can lead to blindness in severe cases [1, 2]. Urinary tract infections (UTI’s) can lead to urethritis, ureteritis, nephritis and cystitis, which can be identified by the brown staining of fur around the rump area, commonly known as ‘wet bottom’, due to incontinence [1]. Reproductive tract infections can cause inflammation and fibrosis causing cystic enlargement of the ovarian bursae, metritis, salpingitis, pyometra, hydrosalpinx and vaginitis in females [1], and prostatitis, orchitis and epididymitis in males [3]. Reproductive tract disease can lead to infertility in both males [3] and females [1, 4].

Despite the importance of chlamydial infection and disease in terms of its effects on wild koala populations, relatively few studies have been conducted to better understand its prevalence, transmission and pathogenesis, for the improvement of treatment and control. Previous studies conducted on the prevalence of infection in wild koala populations have had varying results with some parts of the country reporting high levels of infection (100%), while others have reported none [5–10]. Hospital-based studies have played a major part in data collection relating to infection and disease in wild koalas, due to the cost of capturing and sampling koalas in the wild. Studies involving necropsies performed on koalas presented to wildlife hospitals by wildlife officers and field workers [11–14], have revealed prevalences of infection and disease to be as high as 88% and 21%, respectively. Studies of koalas admitted to wildlife hospitals have also assisted with our understanding of: 1) admission trends and prevalence of chlamydial disease in koalas [15]; 2) the demographics, aetiologies and subsequent outcomes of koalas presented to wildlife hospitals [16, 17]; 3) the relationship between chlamydial load and disease [2]; and 4) the diversity and prevalence of *C*. *pecorum* strains in wild koalas [18].

Whilst studies conducted within wildlife hospitals have contributed to our understanding of chlamydial infection and disease, they use a biased subset of the wild koala population, as only sick and injured koalas are presented for treatment. As such, they do not allow determination of the true prevalence of infection and disease in wild koala populations. This has somewhat been addressed by undertaking free-ranging wild koala population studies. In Victorian koala populations, one study evaluated the prevalence of *C*. *pecorum* (1.4%) and the subsequent genotype of an isolated island population, however, this study was restricted to the urogenital site of female koalas [8]. In another study on Victorian koalas, *C*. *pecorum* was identified in only two of the three populations sampled (41.3%, 25% and 0%) based on swabs of the urogenital site, predominately in female koalas, with males only sampled from one of the three populations [9]. Two free-ranging koala populations were evaluated in Queensland, revealing a significant difference in *C*. *pecorum* infection prevalence of 73% compared to 10%, between the two locations, highlighting the geographical variation in prevalence [6]. The evaluation of clinical disease status in this study resulted in a disease prevalence of 17%, however, this was only based on overt signs, which can significantly underestimate true disease prevalence [6].

Using a standardised and comprehensive veterinary examination, rather than relying on overt signs of disease, in combination with sensitive molecular testing for *C*. *pecorum*, we present a large cross-sectional study of 160 koalas residing in a South East Queensland wild population to gain an accurate insight into koala chlamydial infection and disease prevalence. We analysed samples from koalas, aged between 9 months and 10 years, to determine the prevalence of *C*. *pecorum* infections. We present here important new epidemiological information regarding chlamydial disease in wild koalas.

## Materials and methods

### Animals

Koalas examined in this study (n = 160) were from a population residing in the Moreton Bay region of South East Queensland, Australia between March 2013 and March 2014 (Table 1). As part of a management program for a major infrastructure project in the Moreton Bay region of South East Queensland, koalas were captured for longitudinal monitoring using radio-telemetry and were subjected to comprehensive and standardised veterinary examinations (http://www.tmr.qld.gov.au/Projects/Featured-projects/Moreton-Bay-Rail.aspx). The study area (project centre-point: 27.25° S, 153.02° E) was divided into 6 habitat polygons (regions A–F), and the capture location of each koala was recorded. These regions were not contained and koalas were free to roam between each region. The distance between regions A and F was approximately 10km. The koalas being monitored ranged in age between 9 months and 10 years, as determined by tooth wear class [19] and known or estimated birth dates for some young animals. Young koalas aged 9–13 months were either mother-dependent or recently independent and were considered not to be sexually active. Young koalas aged between 13 months and 2 years of age were mostly independent from their mother and many were sexually active, as evidenced by detection of pregnancy or pouch young during veterinary examinations. All procedures relating to this study were approved by the University of the Sunshine Coast (USC) Animal Ethics Committee (Animal ethics number AN/A/13/80) and by the Queensland Government (Scientific Purposes Permit, WISP11532912).

**Table 1:** Koalas sampled per month, sex and prevalence of *C*. *pecorum* infection and disease.

Month captured/Sampled	# Koalas (Male/Female)	*C*. *pecorum* Infected (n)	Clinically Diseased (n)
January	18 (8/10)	17% (3)	33% (6)
February	11 (3/8)	9% (1)	46% (5)
March	8 (2/6)	88% (7)	25% (2)
April	12 (3/9)	83% (10)	8% (1)
May	14 (5/9)	36% (5)	14% (2)
June	5 (1/4)	0% (0)	60% (3)
July	6 (1/5)	0% (0)	50% (3)
August	8 (5/3)	25% (2)	25% (2)
September	8 (3/5)	63% (5)	25% (2)
October	19 (8/11)	26% (5)	11% (2)
November	35 (20/15)	29% (10)	34% (12)
December	16 (8/8)	6% (1)	25% (4)
**Total**	**160 (67/93)**	**49**	**44**

Number of koalas sampled at each month, their sex, chlamydial infection, as assessed by *C*. *pecorum*-specific qPCR, and clinical disease status, as assessed by physical veterinary examination.

### Veterinary examination and sample collection

Comprehensive and standardised veterinary examinations were performed on all koalas under general anaesthesia to assess their health status. Examinations consisted of a general physical examination and a range of diagnostic techniques to detect most known conditions in koalas, particularly chlamydiosis. These included, ultrasound examination of the bladder, kidneys and reproductive tract, and ultrasound-guided cystocentesis. Biological swabs were collected from the conjunctiva of both eyes and the urogenital sinus (females) and urethra (males) to determine the chlamydial load. Swab samples were then stored at -20°C until analysed. The prevalence of infection and disease in the Moreton Bay koala population was calculated using clinical and biological data collected from the initial veterinary examination of each koala.

### DNA extraction and *Chlamydia pecorum* detection and quantification

DNA was extracted from the ocular and urogenital swabs using a QIAmp DNA mini kit as per manufacturer’s instructions (Qiagen). Extracted DNA was then screened for *C*. *pecorum* chlamydial presence and load using quantitative real-time PCR (qPCR). The forward primer: 5’ AGTCGAACGGAATAATGGCT 3’, and the reverse primer: 5’ CCAACAAGCTGATATCCCAC 3’ were used for targeting a 204bp fragment of the *C*. *pecorum* 16S rRNA gene. All procedures were as previously described by Marsh *et al*. (2011) [20] except for the PCR mixture containing 1 x Quantitect SYBR Green PCR mastermix (Qiagen) and 10 μM primers (Sigma) made up to a final volume of 15 μl with PCR-grade water and an initial denaturation of 15 minutes at 94°C. All reactions were performed in duplicate and samples of ≥ 50 copies/μL were considered positive. All reactions were carried out on a Rotor-Gene Q 5-plex HRM platform (Qiagen).

### *Chlamydia pecorum* ompA genotyping

Outer membrane protein A (ompA) sequencing was performed on a sub-set (19/49) of *C*. *pecorum* positive koalas to determine the genetic diversity represented within our population. *C*. *pecorum* positive DNA, as determined by qPCR from either ocular or UGT swab samples, was used as template for conventional PCR amplification. The forward primer: CpeNTVD3 5’-GTTCTTTCTAACGTAGC-3’, and the reverse primer: CpeNTVD4 5’-TGAAGAGAAACAATTTG-3’ were used to amplify the VD3 and VD4 regions of the ompA gene targeting the 359bp fragment located at the 670 – 1028bp region of the full length ompA gene. Each PCR reaction contained 1 μL of gDNA template added to a mastermix containing, 12.5μL of 2x HotStar Taq (Qiagen), 0.3mM of each forward and reverse primer and molecular grade water making a final volume of 25μL per reaction. For PCR amplification, there was an initial denaturation at 95°C for 5 mins, then 40 cycles of denaturation at 95°C for 30 secs, primer annealing at 46°C for 30 secs, primer extension at 72°C for 30 secs, followed by a final extension at 72°C for 5 mins. All ompA sequences were determined by direct sequencing of the PCR product using CpeNTVD3/CpeNTVD4 performed by Macrogen Inc. (Korea).

### Statistical analysis

For all models we followed standard procedures for data exploration [21] and ensured there were no outliers in the variables, as well as no collinearity between the explanatory variables.

To model the probability of a koala being infected (presence of absence) or being diseased (presence or absence) as a function of the covariates, a binary logistic regression, using Generalized Linear Models (GLM) was used. We coded infection or disease as a binary response variable (1 = presence, 0 = absence). We also used a subsample to model the disease as a function of genotype. We fitted the binary logistic regressions using the logit link function in the binomial family of GLM using R version 3.4.0 [22]. We used Akaike Information Criteria to assess the importance of the explanatory variables and identified the minimum adequate model using the likelihood ration test. We then predicted the probability of infection or disease using the explanatory variables identified as most significant within the model.

To determine drivers of *C*. *pecorum* infectious load, after data exploration identified zero inflation and a high variability in the chlamydial load, we utilised a Bayesian approach with a negative bionomial general linear model. The Bayesian analysis framework was adopted with Markov chain Monte Carlo (MCMC), which is essentially a simulation technique to obtain the distribution of each parameter in a model [21]. To fit the model, MCMC was applied using JAGS [23] via the package R2jags[24]. We used a burn-in of 50,000 iterations, three chains, a thinning rate of 10,000 iterations for each posterior distribution. Diffuse normal priors were used for regression parameters and diffuse uniform priors for the standard deviation parameters. Mixing of the chains was good.

## Results

**Fig 1:**
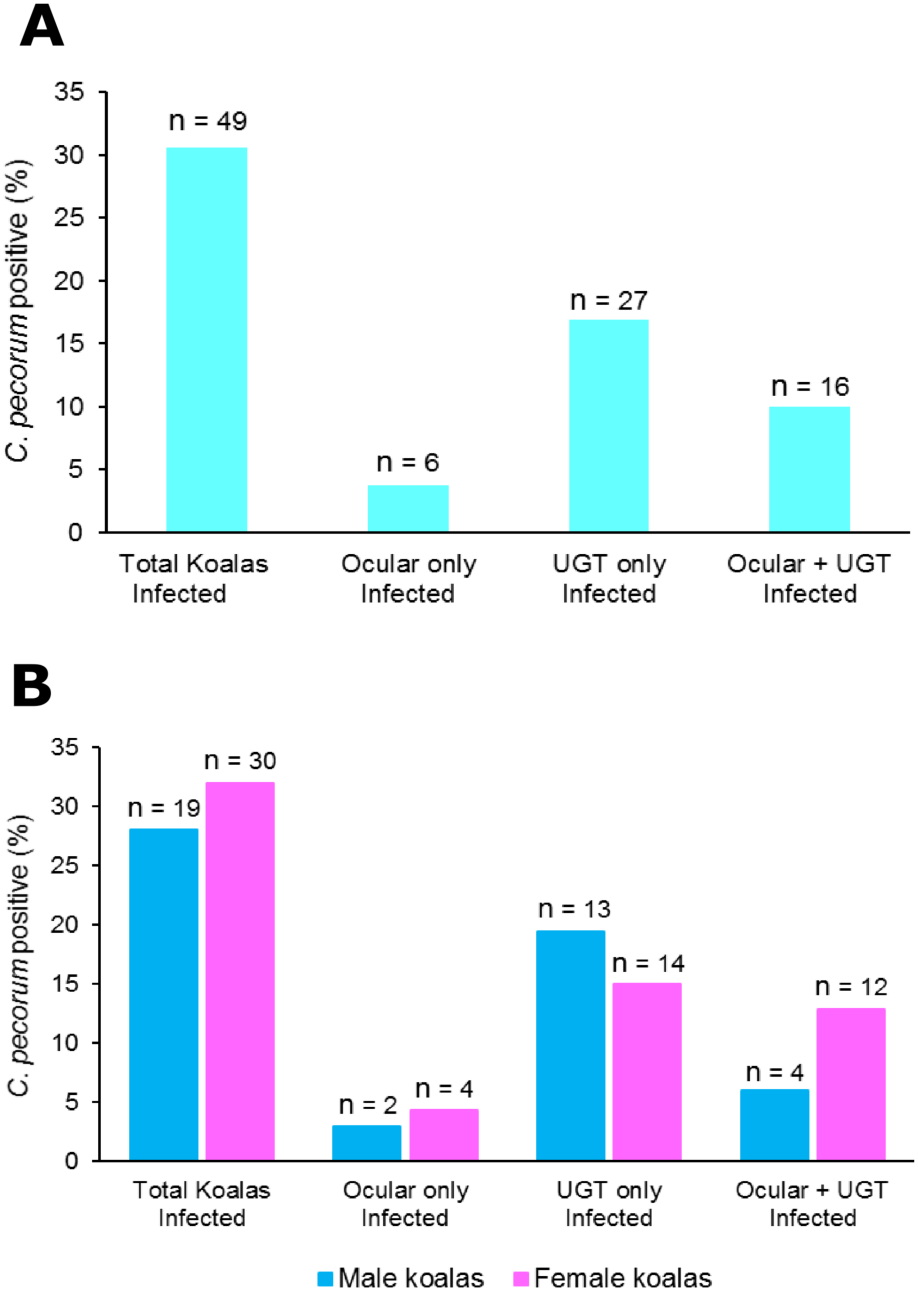
A: Prevalence of chlamydial infection, as assessed by *C*. *pecorum*-specific qPCR, in 160 koalas from a free-ranging population in South East Queensland. The prevalence of *C*. *pecorum* infection identified at the ocular, urogenital (UGT) or ocular plus urogenital (UGT) site based on qPCR *C*. *pecorum* load. B: Prevalence shown in panel A separated by sex.

We used a quantitative *C*. *pecorum*-specific PCR assay to measure the infection levels in this wild koala population over a 12-month period (Table 1). In this particular peri-urban population, 31% (49) of the 160 koalas sampled were infected with *C*. *pecorum*. Of these, 4% (6) had an ocular-only infection, 17% (27) had a urogenital-only infection and 10% (16) had infections at both the urogenital and ocular sites (Fig 1A).

### Similar prevalence of infection found in both males and females

We found similar overall infection prevalence in both sexes, with 28% (19/67) of males infected and 32% (30/93) of females infected (Fig 1B). The anatomical site of the infections was also similar between the sexes; 3% versus 4% for ocular-only infections; 19% versus 15% for urogenital-only infections; 6% versus 13% for two-site infections, for males versus females respectively.

### Prevalence of infections by age of the koala

**Fig 2:**
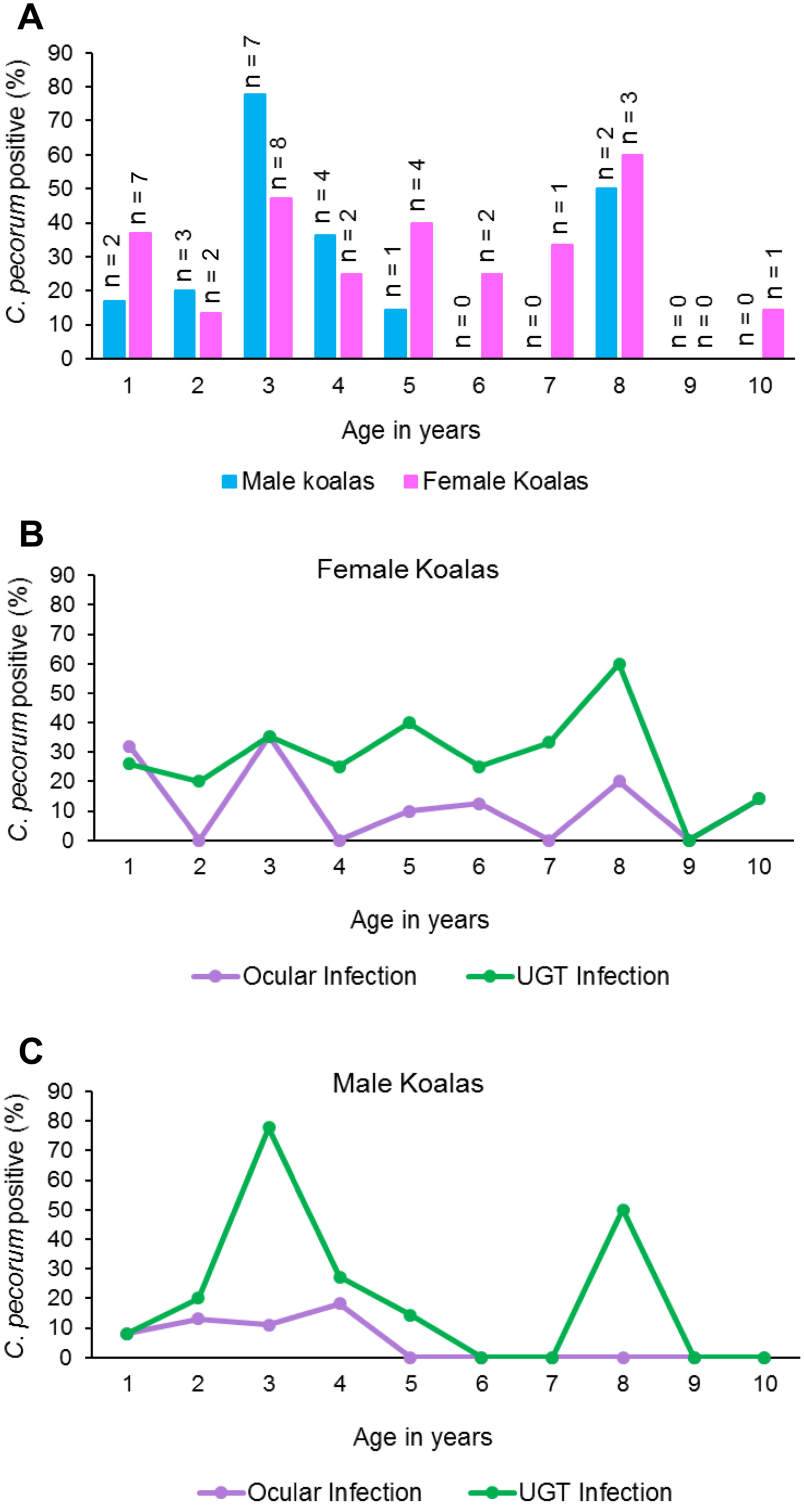
A: Prevalence of *C*. *pecorum* infection in male versus female koalas between the ages of 9 months and 10 years based on being qPCR positive. B: Prevalence of *C*. *pecorum* infection per site of infection in female koalas aged between 9 months and 10 years based on being qPCR positive. C: Prevalence of a *C*. *pecorum* infection per site of infection in male koalas aged between 9 months and 10 years based on being qPCR positive.

Interestingly, 29% (9/31) of young (<2 years) koalas were *C*. *pecorum* positive. However, this rate differed between the sexes with 17% (2/12) of males infected compared to 37% (7/19) of females infected (Fig 2A). The prevalence of infection for koalas aged 9–13 months (considered pre-sexually mature) was 27% (4/15) and similarly 31% (5/16) for koalas aged 13 months-2 years (Table 2). We observed a higher prevalence of *C*. *pecorum* infection in koalas aged 3 years (58%;15/26) (Fig 2A). For females, the prevalence of infection for 3 to 8 year-old koalas remained similar, then a slightly lower prevalence for koalas aged 10 years (Fig 2A). However, in male koalas, we observed a lower prevalence of infection after age 3 years with only 2 males (15%) infected (at age 8) between the ages of 6 and 10 (Fig 2A).

**Table 2:** Koala age, sex and prevalence of *C*. *pecorum* infection and disease.

Age captured/Sampled	# Koalas (Male/Female)	*C*. *pecorum* Infected (Male/Female)	Clinically Diseased (Male/Female)
1 - (9–13 months)	15 (4/11)	27% (1/3)	20% (1/2)
1 - (13 months—2 years)	16 (8/8)	31% (1/4)	19% (2/1)
1 - (9 months—2 years)	31 (12/19)	29% (2/7)	19% (3/3)
2	30 (15/15)	17% (3/2)	13% (1/3)
3	26 (9/17)	58% (7/8)	38% (4/6)
4	19 (11/8)	32% (4/2)	32% (2/4)
5	17 (7/10)	29% (1/4)	29% (2/3)
6	11 (3/8)	18% (0/2)	27% (1/2)
7	5 (2/3)	20% (0/1)	40% (1/1)
8	9 (4/5)	56% (2/3)	44% (2/2)
9	3 (2/1)	0% (0/0)	33% (0/1)
10	9 (2/7)	11% (0/1)	33% (1/2)
**Total**	**160 (67/93)**	**19/30**	**17/27**

Number of koalas sampled per age, their sex, chlamydial infection, as assessed by *C*. *pecorum*-specific qPCR, and clinical disease status, as assessed by physical veterinary examination.

Differences in the anatomical site of the infection across the age and sex of the koalas were also noted. Young female koalas (<2 years of age) had a similar prevalence of ocular and UGT infection (32% and 26% respectively), then we observed a slightly higher prevalence of infections (35%), at the ocular and UGT site, in koalas aged 3 years (Fig 2B). The prevalence of a UGT infection then remained similar for females aged 4–8 years, before observing a lower prevalence in koalas aged 9 and 10 years (Fig 2B). Although the prevalence of ocular infections was also observed to be similar between koalas ages 4–10 years, they were at a lower prevalence (Fig 2B). In contrast, a different pattern emerged for male koalas, with young (<2 years of age) male koalas having an 8% prevalence of infection for both the ocular and UGT sites (Fig 2C). Interestingly, we found that 78% of male koalas aged 3 years had a UGT infection, we then observed a lower prevalence of UGT infection in male koalas aged 4 and 5 years then 0% at age 6 years (Fig 2C). The prevalence of a UGT infection in male koalas was then observed to be 50% in 8 year old animals, before we again observed a prevalence of 0% at ages 9 and 10 years (Fig 2C). The highest prevalence of ocular infection amongst male koalas was observed at age 4 years (18%), then no ocular infections were observed in male koalas between the ages of 5 and 10 years (Fig 2C).

We used a modelling approach to examine the multi-factional relationships of our parameters on chlamydial infection and disease. The model that best explained the prevalence of chlamydial ocular infections included age and sex, but not geographical location. We found that, while allowing for any effects from the other parameters, the prevalence of ocular infections in both male and female koalas decreased with age, and that the prevalence of chlamydial ocular infections was higher in young females than in young males. However, this sex effect disappeared in older koalas (S1 Fig; S1 Table). For UGT infections, location, sex and age did not explain any of the variation in the chlamydial prevalence. There were no important changes in *C*. *pecorum* UGT infections that can be attributed to the sex or the age of the koala (S2 Fig).

### Chlamydial load was higher in koalas with no clinical disease

The chlamydial load (as measured by quantitative PCR) for ocular infected male koalas ranged from 73 to 4,247 copies/μL (median 435) and in female koalas ranged from 76 to 7,923 copies/μL (median 388) (Table 3). The chlamydial load for UGT infected male koalas ranged from 58 to 263,284 copies/μL (median 739) and in female koalas ranged from 50 to 1,094,553 copies/μL (median 1,461) (Table 3). The chlamydial load for koalas who were *C*. *pecorum* infected with signs of clinical disease was significantly lower (ranged from 73 to 9,605 copies/μL; median 725.5; Table 3) compared to koalas who were *C*. *pecorum* infected but with no signs of clinical disease (ranged from 50 to 1,094,553 copies/μL; median 988; Table 3). Interestingly, we found that the six highest chlamydial loads, for both the ocular site (1 male; 5 females) and UGT site (3 males; 3 females), were in koalas who were *C*. *pecorum* infected but had no signs of clinical disease. While these individual relationships are clearly important, when we used a Bayesian approach with a negative binomial general linear model, we found that neither sex, age, geographical location, nor disease status were important drivers of chlamydial load, at either the ocular or UGT sites.

**Table 3:** Chlamydial load for ocular and UGT infected koalas showing their sex, age and clinical disease status and ompA genotype.

Koala Identification #	Male/Female	Koala Age	Ocular chlamydial Load copies/μL	UGT chlamydial Load copies/μL	Clinically Diseased	Ocular ompA Genotype	UGT ompA Genotype
182	Male	2	73	712	Diseased	E'	E'
143	Male	3	0	739	Diseased	G	
117	Male	8	0	1795	Diseased		
102	Male	4	100	7940	Diseased		
159	Male	3	0	9605	Diseased		F
305	Male	9–10 months	1427	0	Diseased		
107	Male	3	0	58			E'
292	Male	3	0	69			
168	Male	4	0	78			
4	Male	2	0	91			
42	Male	2	108	150			
192	Male	8	0	225			
196	Male	5	0	630		G	
110	Male	1.5	0	2898			E'
12	Male	3	0	8481			
222	Male	3	0	13161			E'
17	Male	3	435	29000			
255	Male	4	0	263284			E'
122	Male	4	4247	0			
103	Female	1.5	178	304	Diseased	E'	E'
158	Female	3	0	465	Diseased		E'
138	Female	8	0	1263	Diseased		E'
191	Female	5	0	1356	Diseased		E'
171	Female	4	0	1422	Diseased		
243	Female	1.1	0	1513	Diseased		E'
303	Female	3	0	6307	Diseased		
169	Female	3	182	0	Diseased		
242	Female	1.1	76	0	Diseased	E'	
46	Female	7	0	50			
173	Female	2	0	52			
48	Female	1.5	175	60			
51	Female	3	100	115			
31	Female	5	195	195			
124	Female	6	0	569		G	
113	Female	2	0	775			G
39	Female	8	130	988			
16	Female	5	0	1500			
21	Female	8	0	1983		G	
7	Female	1.5	2135	2500			
14	Female	4	0	3251			
5	Female	3	27923	3354			
27	Female	11–12 months	4811	5268		G	G
13	Female	5	0	5425			
23	Female	3	352	6828			
30	Female	10	14268	35400			
10	Female	3	1623	120000			
9	Female	6	1406	1094553			
111	Female	1.5	424	0		G	
126	Female	3	736	0			

Number of koalas *C*. *pecorum* positive, as assessed by *C*. *pecorum*-specific qPCR, as per their sex, age, ocular a UGT chlamydial load, clinical disease status, as assessed by physical veterinary examination and ompA genotype.

### Diversity of ompA genotypes

A sub-set of *C*. *pecorum* infected koalas (19) were genotyped using ompA gene sequencing to identify the MOMP strains circulating within this population (Table 3). The genotype was identified from either the ocular site (6), UGT site (10) or both (3 koalas). The most prevalent *C*. *pecorum* ompA genotype identified was genotype E’ (58%; 11/19), followed by genotype G (37%; 7/19) and genotype F (5%; 1/19). However, when we examined koalas that were PCR positive with no clinical disease (10), 40% (4/10) were genotype E’ and 60% (6/10) were genotype G. This differed slightly to what we observed in koalas who were infected and clinically diseased (9) with 78% (7/9) genotype E’, 11% (1/9) genotype G and 11% (1/9) genotype F. When we examined the spread of genotypes between the sexes, we found similar results with 62.5% (5/8) versus 55% (6/11) for genotype E’ and 25% (2/8) versus 45% (5/11) for genotype G, for males versus females respectively. We did however, find potentially relevant differences in the genotypes present in ocular versus UGT sites. The most prevalent genotype identified at the ocular site was genotype G (67%; 6/9) compared to 77% (10/13) genotype E’ at the UGT site. All three koalas genotyped at both the ocular and UGT sites had the same ompA strain present.

**Fig 3:**
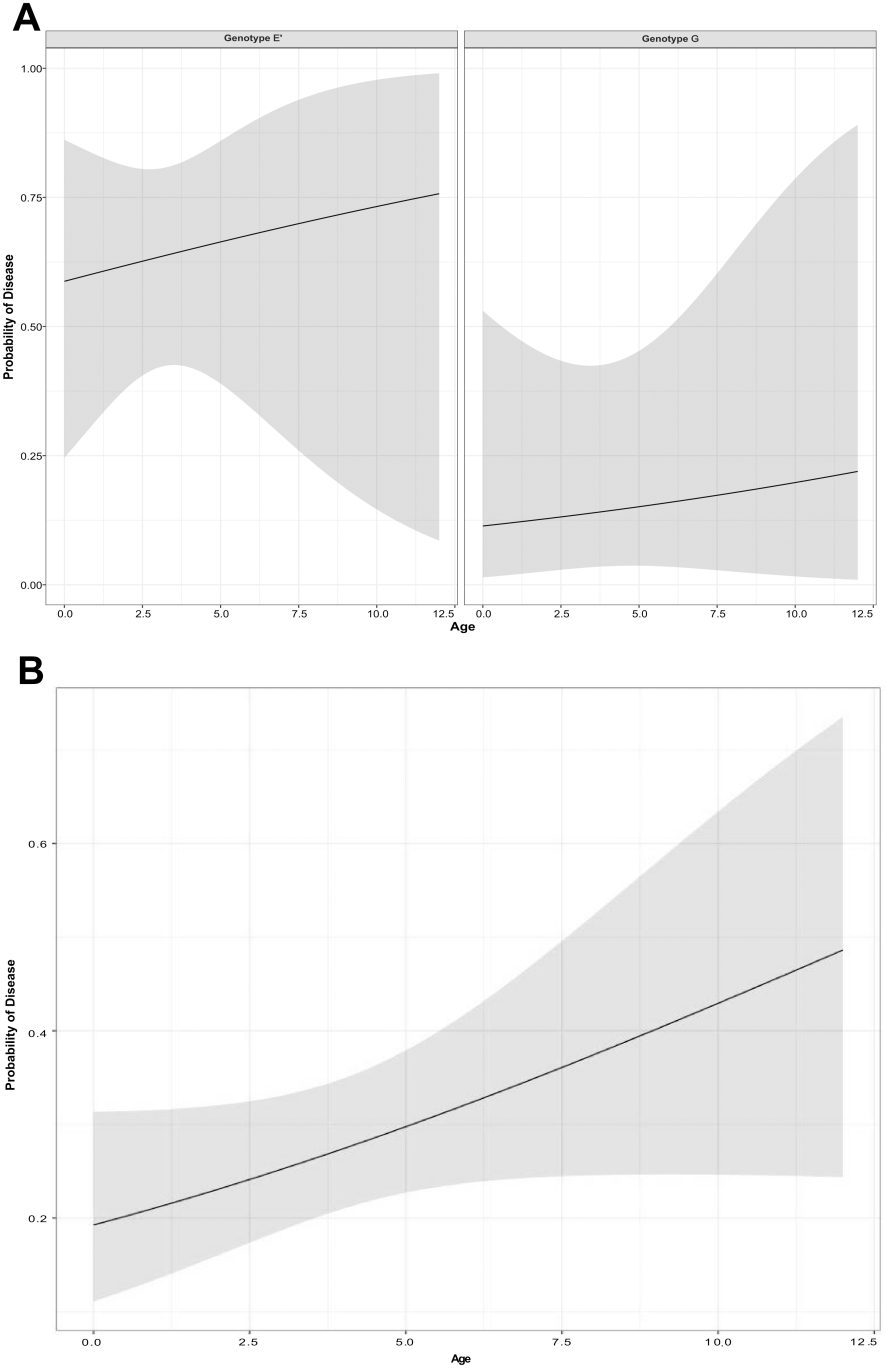
A: Fit of the general linear model for the probability of disease with increasing age and separated by chlamydial genotype. B: Fit of the general linear model for the probability of being diseased with increasing age.

When we investigated the multi-factional relationships using general linear models, we found that the probability of a koala having disease could be partly explained by the infecting genotype. Koalas infected with genotype E’, were more likely to have disease than koalas infected with genotype G (Fig 3A; S2 Table).

### Geographical distribution of infections across the population sub-regions

Our study population was defined by an area that was located along and beside the corridor for construction of a transport infrastructure project and was arbitrarily divided into six regions (A to F). Because a large percentage of the koalas analysed were found in our outermost regions A and F, 65 (41%) and 61 (38%) out of the total 160 koalas respectively, we compared the prevalence of infection between these two “end” regions, which are only 10km apart. The prevalence of infection (as defined by qPCR positivity) was 26% (17/65) in region A and 39% (24/61) in region F. Urogenital tract infections were more common than ocular infections, in both regions, with a UGT infection prevalence of 26% (17/65) in region A and 28% (17/61) in region F compared to the ocular infection prevalence of 11% (7/65) in region A and 20% (12/61) in region F.

### Urogenital tract disease was prevalent in this population

We performed detailed veterinary health examinations, allowing us to assess both clinical and sub-clinical chlamydial disease. The clinical and sub-clinical diseases identified within our population group were, cystitis, reproductive disease and kerato-conjunctivitis. Twenty-eight % of the koalas studied (44/160) had chlamydial disease that was evident either clinically (48%, 21/44) or sub-clinically (52%, 23/44). Ocular disease was less prevalent (8/44) compared with UGT disease (37/44), which included 57% urinary tract disease and 39% reproductive tract disease.

**Fig 4:**
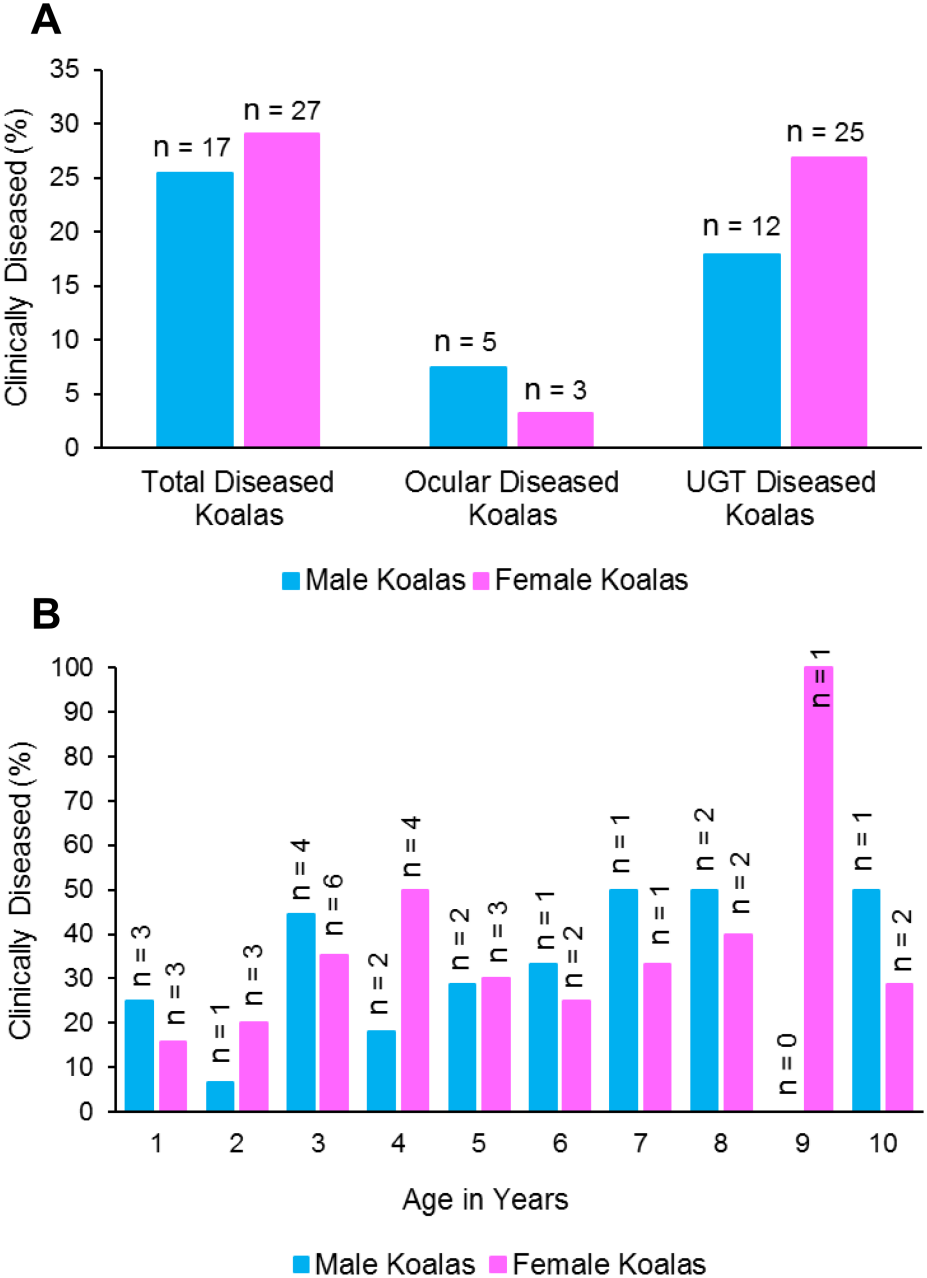
A: Prevalence of disease amongst male versus female koalas. The prevalence of disease identified at the ocular or urogenital tract site is based on veterinary examination. B: Prevalence of chlamydial disease in koalas between the ages of 9 months and 10 years based on veterinary examination.

Males and females had a similar prevalence of disease (25% versus 29% respectively) however, the anatomical site of the lesions varied, with males having a higher prevalence of ocular disease than females (7% versus 3%) and a lower prevalence of UGT disease than females (18% versus 27%) (Fig 4A). When we analysed the koala age at which disease became evident, we found that 19% (6/31) of koalas became diseased from a young age (<2 years), with the same prevalence observed in both males and females (Fig 4B). The prevalence of disease in koalas aged 9–13 months was 20% (3/15) and 19% (3/16) for koalas aged between 13 months and 2 years. Disease prevalence increased to 38% (10/26) in koalas by 3 years of age and then remained relatively constant, for both sexes, into older aged animals (Fig 4B). The model that best explained the presence of disease included age, but not sex nor geographical location. We found that the probability of disease in a koala increased with increasing age (Fig 3B; S3 Table).

**Fig 5:**
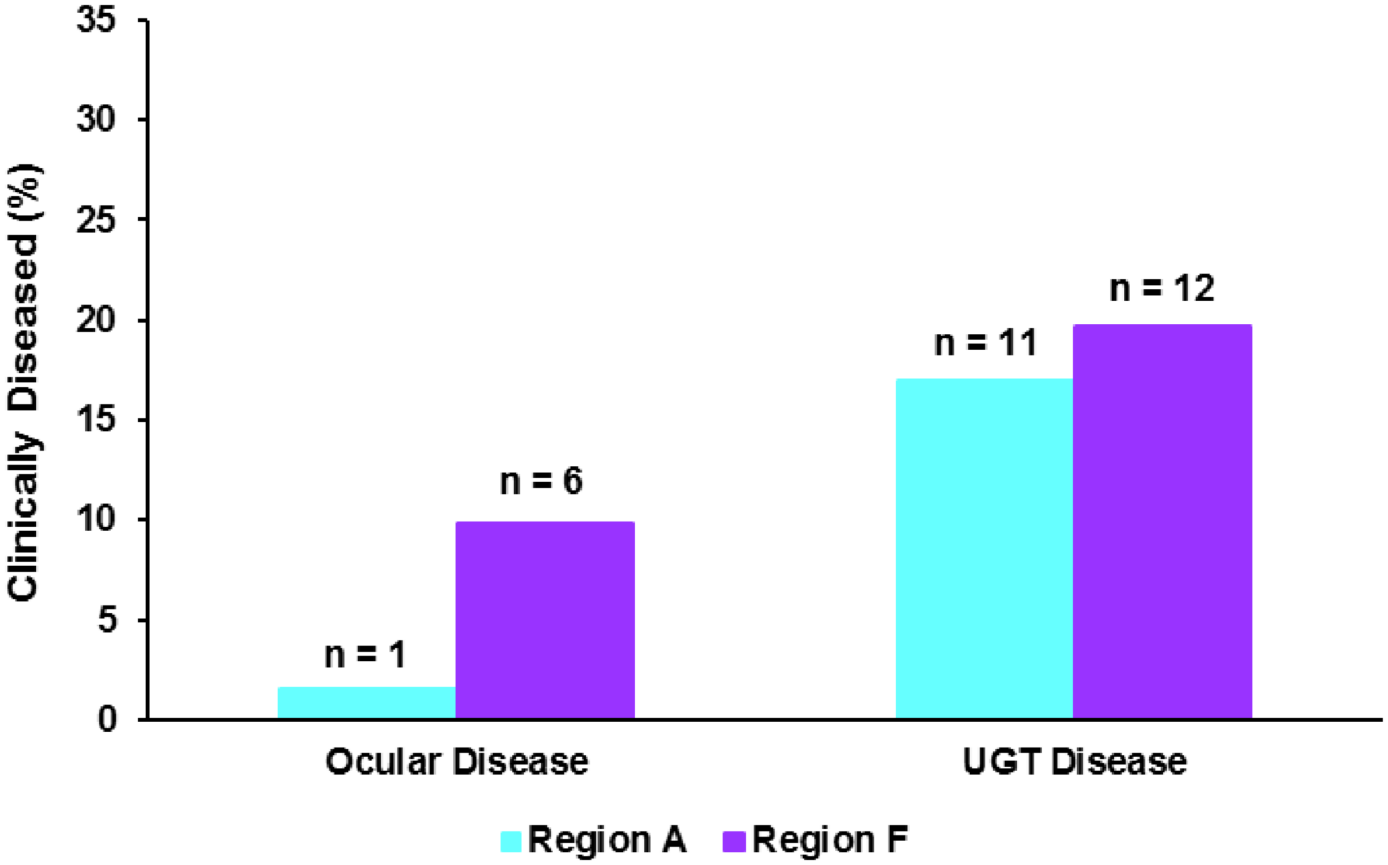
Prevalence of chlamydial disease at the ocular and urogenital tract site in koalas residing in the end regions of our study, A versus F.

Finally, we compared the prevalence of chlamydial disease seen in the geographical end regions of our study population (Fig 5). Similar to our observations of chlamydial infection prevalence, as determined by qPCR, we observed a higher prevalence of urogenital tract disease than ocular disease in both regions, with a UGT disease prevalence of 17% (11/65) in region A and 20% (12/61) in region F compared to ocular disease prevalence of 2% (1/65) in region A and 10% (6/61) in region F (Fig 5).

### Limitations of our study

While this is one of the largest and most detailed studies of chlamydial infection and disease in a single koala population, it does have some limitations. We sampled the 160 koalas over a 12-month period as it was impossible to sample all animals at a single moment in time. Therefore, there may be some effects due to differences in the time of the year that individual animals were sampled.

## Discussion

Koala populations in most parts of Australia, particularly in South East Queensland, are declining, with the main contributing factors being, 1) injuries sustained from road trauma [25, 26]; 2) infectious diseases, such as *Chlamydia* [6, 7, 9] and koala retrovirus (KoRV) [27–30]; 3) dog attacks [31]; and 4) habitat destruction [32]. *Chlamydia* causes disease in the koala with studies showing koalas to be infected with several chlamydial species, including, *C*. *pecorum*, *C*. *pneumoniae* and *Chlamydia*-like organisms [7, 33] however, it is *C*. *pecorum* that has been shown to be more prevalent and more pathogenic [6, 7, 34]. Our study not only directly detected *C*. *pecorum* infections and overt clinical disease in koalas but also, by using ultrasound and performing cystocentesis during the veterinary examination, we could further identify sub-clinical disease, which may otherwise have remained undetected. Our data show that up to 50% of disease is sub-clinical and would be missed without the use of veterinary diagnostic aids, such as ultrasound and cystocentesis. The importance of comprehensive veterinary examinations for the identification of sub-clinical disease has also been highlighted by Patterson *et al*. (2015) and Speight *et al*. (2016) [9, 11].

Our study resulted in several major findings which have added valuable information and insight into the epidemiology of *C*. *pecorum* infection in free-ranging koala populations. In our population, there was 31% overall prevalence of *C*. *pecorum* infection and a similar prevalence of clinically significant disease (28%). Previously reported prevalences of chlamydial infections in wild koala populations have ranged widely, as follows: 1) Speight *et al*. (2016) reported an 88% prevalence of a *C*. *pecorum* infection in South Australia [11]; 2) in Victoria, Patterson *et al*. (2015) reported 0%, 25% and 41% (at three separate locations) [9]; 3) and previously in Queensland, Jackson *et al*. (1999) reported 10% and 73% (separate locations) [6], Kollipara *et al*. (2013) reported 45% [18], Deveraux *et al*. (2003) reported 52% [7] and White and Timms (1994) reported 39%, 49%, 53% and 61% (separate locations) [35]. In our South East Queensland population, UGT infections were much more prevalent than ocular infections; 27% compared to 14% respectively, which was consistent with several other studies [5, 6, 9, 18, 35].

Although *C*. *pecorum* is a sexually transmitted infection [36], this does not explain the presence of both chlamydial infection and disease that we found in young koalas that were not yet sexually mature. While it has been suggested that mother to young transmission might occur during birth, through pap feeding or through the constant and close contact between a mother and her joey, there has been no published evidence, to date, to support this theory. Our data provide compelling evidence that this occurs, and is an important route of infection. We also observed that the prevalence of infection in dependent male juvenile koalas was lower at the urogenital site compared with dependent female juveniles. This is likely to be due to the relatively higher risk of urinary tract infection occurring between an infected mother and female joey, compared with male joeys, due to anatomical differences. Ocular infections were more common in koalas less than 3 years of age, and occurred in dependent young (sexually immature), suggesting that maternal to joey infection is a common route of ocular infection. Together, our results provide evidence to support the hypothesis of a mother to joey transmission route, with both ocular and UGT sites being infected equally at this age. Mother to baby transmission has also been shown in humans with *C*. *trachomatis*, where babies born to vaginally infected mothers developing conjunctivitis [37–39]. After this initial mother to young direct contact transmission in koalas, our data suggest that the UGT site then becomes the primary site of new infections, presumably due to sexual transmission between males and females [36].

In this study, we also provided a veterinary analysis of clinical disease in a wild koala population. Our findings of 28% overall prevalence of clinically significant disease was high compared to 17% reported in a previous South East Queensland wild koala study, however this study only evaluated overt disease [6]. We found that only half (48%) of the clinically affected koalas displayed overt clinical signs of disease with the remaining (52%) diagnosed with sub-clinical disease detected by comprehensive veterinary examination. This highlights the likelihood of significant underestimation of disease prevalence in surveys relying upon overt clinical signs alone.

Our finding of a higher prevalence of disease at the UGT site (23%) compared to the ocular site (5%) generally reflected qPCR findings at those sites. Although the chlamydial load varied for both the UGT site (50–1,094,553 copies/μL) and the ocular site (73–27,923 copies/μL) overall, the chlamydial load was also higher at the UGT site for both male and female koalas. Disease prevalence varied somewhat in different age groups, but was at significant levels from early age, through to old age, with a trend for higher disease prevalence as age increased. In comparison, in male koalas the prevalence of infection (verses disease) was lower with increasing age. In females, we observed less variation in prevalence of infection between age-groups, except in aged female koalas (9–10 years-age) in which the prevalence of infection was low. Despite these findings, statistically we found that UGT infection prevalence was not impacted by sex or age (S2 Fig).

Another significant finding from this study, was that while the prevalence of infection and disease were similar (31% and 28% respectively) they were not always seen together. Of the 49 *C*. *pecorum* infected and 44 diseased koalas, we only identified an active *C*. *pecorum* infection combined with a clinical diseased state in 15 (6 males, 9 females) koalas. This is not completely unexpected as similar findings have also been reported by Wan *et al*. (2011) who observed high levels of chlamydial shedding at the UGT site, of some koalas, in the absence of clinical disease [2] and by Patterson *et al*. (2015) who observed diseased states in the absence of a chlamydial PCR positivity in a Victorian koala population [9]. In addition, the observation of higher chlamydial loads in *C*. *pecorum* infected koalas with no signs of clinical disease (50–1,094,553 copies/μL) compared to *C*. *pecorum* infected koalas with clinical disease (73–9,605 copies/μL) would also indicate that chlamydial load alone does not seem to drive disease. This is further supported by our statistical analysis showing that neither sex, age, location or disease status are drivers of chlamydial load. There are several other possibilities that might contribute to *C*. *pecorum* PCR negative but clinically diseased koalas, including the presence of other chlamydial species, other viral infections, including infection with the koala retrovirus, KoRV. Indeed, KoRV strain B has been reported to be a significant contributing factor to the development of chlamydial disease [30]. However, the presence of clinical disease in the absence of PCR positivity indicates that koalas may resolve their *C*. *pecorum* infection, or at least temporarily cease shedding of organisms, without resolving clinical signs or pathological changes.

Our findings, relating to the prevalence of ompA genotypes circulating within our study area, add insight into the pathogenesis of chlamydial disease in wild koala populations. Interestingly, although the most prevalent ompA genotype identified within this sub-section of our population group was genotype E’, with 58% (11/19), this varied depending on the site of infection and the presence or absence of clinical disease. Although, genotype E’ was the most prevalent strain identified at the UGT site with 77% (10/13), genotype G was the most prevalent strain identified at the ocular site with 67% (6/9) of *C*. *pecorum* infected koalas. Similarly, it was interesting that *C*. *pecorum* infected koalas with clinical disease were predominantly infected with genotype E’ (78%; 7/9) whereas, *C*. *pecorum* infected koalas, that had not progressed to a clinical disease state, were predominantly infected with genotype G (60%; 6/10). The differences found within ompA genotypes were interesting and suggest that the site of infection and progression to disease could be influenced by the *C*. *pecorum* strain infecting the koala. We found that although we identified koalas with clinical disease infected with genotypes E’, G and F, statistically, koalas residing within our population group, had a higher probability of progressing to a clinical diseased state when infected with genotype E’. The identification of different ompA genotypes and their potential influence on the pathogenesis of disease within the koala has added valuable information for targeting strains in the development and implementation of an anti-chlamydia vaccine.

This study was unique in its analysis of a significant proportion of a natural population of koalas, both male and female, ranging in age (9 months—10 years) with respect to chlamydial infection and disease. It reinforces the importance of thorough veterinary investigation to appropriately diagnose both clinical and sub-clinical disease. Importantly, we have identified that young, pre-sexually active, koalas are infected with *C*. *pecorum*, providing compelling evidence of mother to joey transmission. The effective management of chlamydial disease to improve population viability in at-risk and declining koala populations, particularly using vaccination and treatment, relies upon a good understanding of chlamydial epidemiology and pathogenesis. Our study has provided important new information, but additional investigation of chlamydial dynamics and pathogenesis at the population level is warranted.

## Supporting information

S1 Fig Fit of the GLM for the probability of being infected at the ocular site with increasing age, separated by sex (females and males). (TIF)

S2 Fig Fit of the GLM for the probability of being infected at the UGT site with increasing age, separated by sex (females and males). (TIF)

S1 Table Estimated regression parameters, standard errors, z-value, and the AIC for the GLM with sex and age explaining the variation for chlamydial infections at the ocular site. (PNG)

S2 Table Estimated regression parameters, standard errors, z-values, and the AIC for the GLM with genotype the variation for chlamydial disease. (PNG)

S3 Table Estimated regression parameters, standard errors, z-values, and the AIC for the GLM with age explaining the variation for chlamydial disease. (PNG)
