# Supplementary figures and images for: Epidemiology of chlamydial infection and disease in a free-ranging koala (Phascolarctos cinereus) population
Source: PLoS One. 2017 Dec 27;12(12):e0190114. doi: 10.1371/journal.pone.0190114 (PMC5744985; doi:10.1371/journal.pone.0190114)

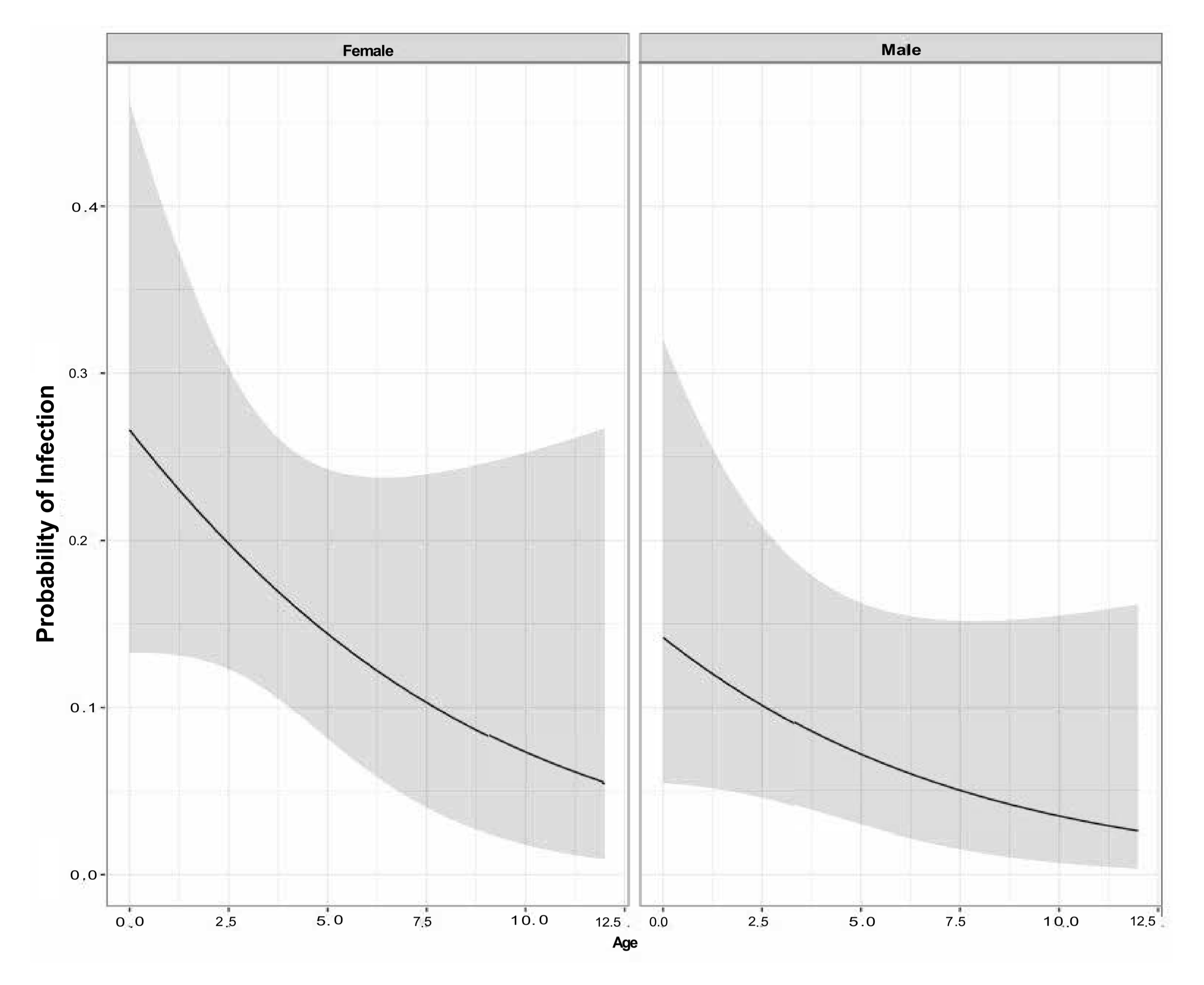

Supplement: S1 Fig — (TIF) [file pone.0190114.s001.tif]

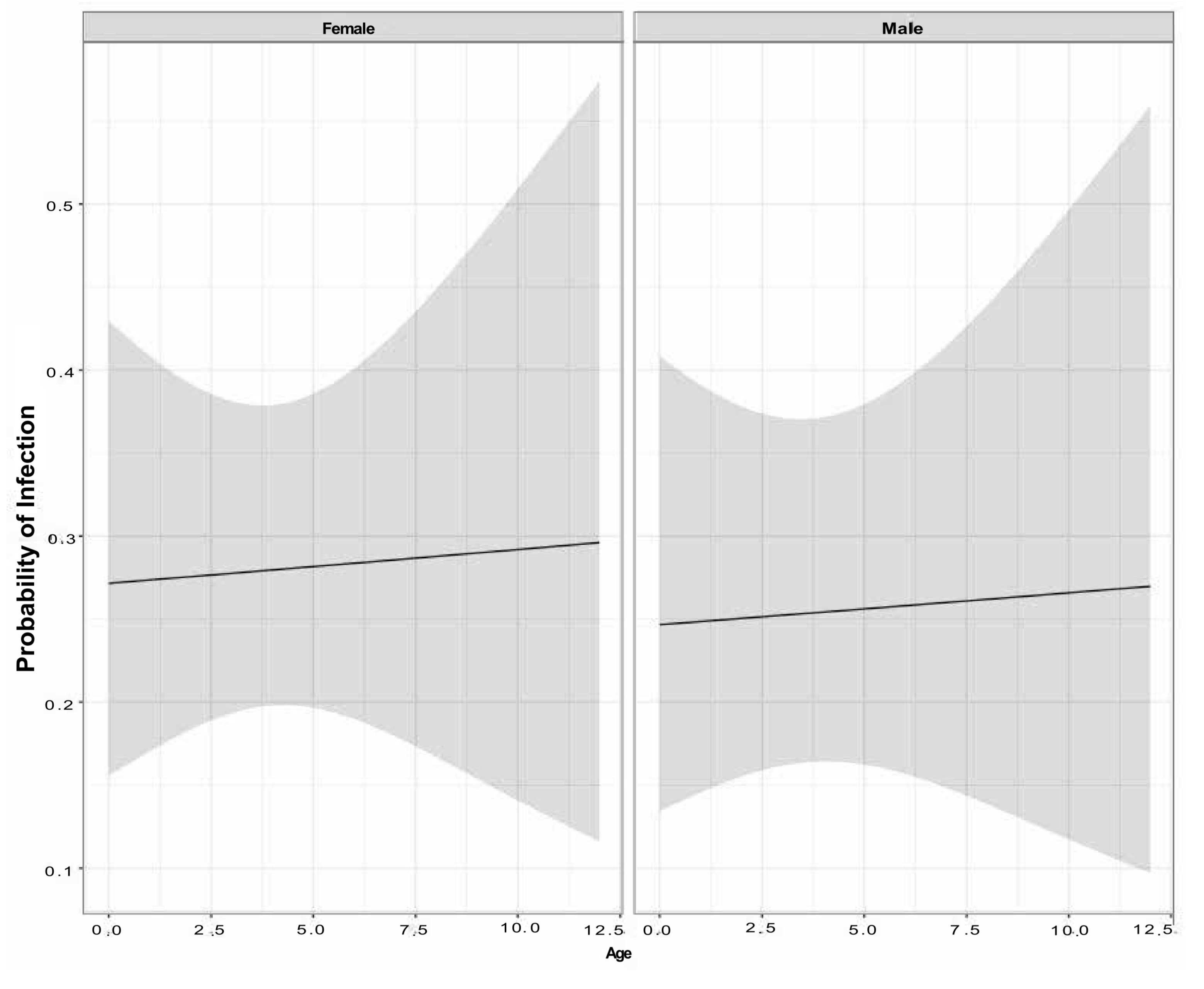

Supplement: S2 Fig — (TIF) [file pone.0190114.s002.tif]

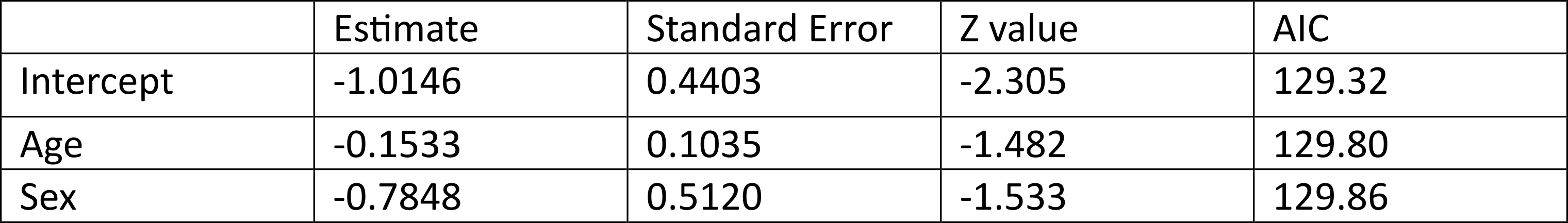

Supplement: S1 Table — (PNG) [file pone.0190114.s003.png]

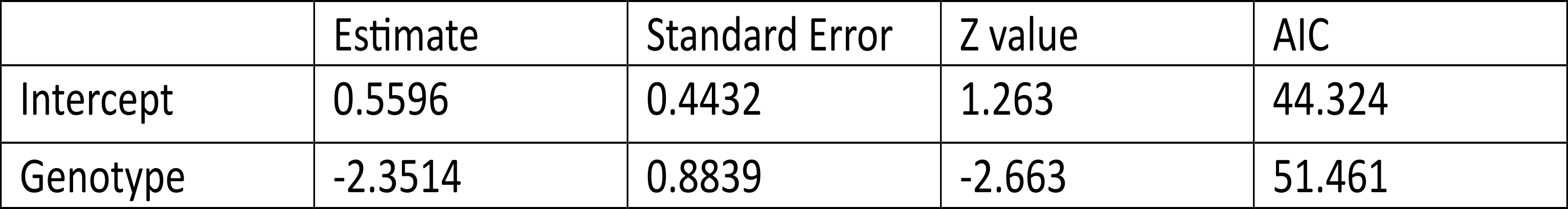

Supplement: S2 Table — (PNG) [file pone.0190114.s004.png]

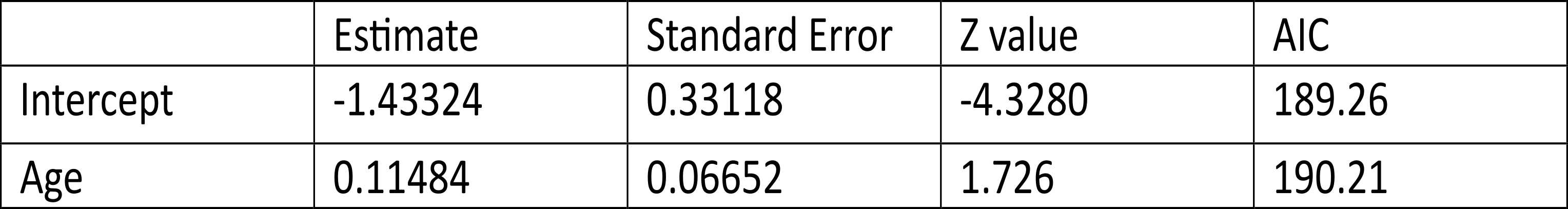

Supplement: S3 Table — (PNG) [file pone.0190114.s005.png]
